# The de Winter–Wellens Continuum: Evidence of Dynamic Bidirectional Coronary Collateral Flow in Acute Coronary Syndrome

**DOI:** 10.1016/j.jaccas.2026.107531

**Published:** 2026-03-24

**Authors:** Jan Mohd Sheikh, Khawar Achakzai, Sameer Purra, Aamir Rashid, Mohd Iqbal Dar, Imran Hafeez, Aijaz Ahmed Lone, Hilal Ahmed Rather, Syed Bilal

**Affiliations:** Department of Cardiology Sheri-Kashmir Institute of Medical Sciences Srinagar, Jammu and Kashmir, India

**Keywords:** acute coronary syndrome, cardiovascular disease, circulation, coronary angiography, electrocardiogram, hypertension, left ventricle, myocardial ischemia

## Abstract

**Background:**

The evolution of a de Winter pattern to a Wellen pattern in acute coronary syndrome is a rare occurrence; both signify critical left anterior descending artery (LAD) lesion and warrant urgent early percutaneous coronary intervention (PCI).

**Case Summary:**

Electrocardiogram (ECG) of a 55-year-old man with retrosternal discomfort demonstrated a de Winter pattern that transiently normalized, leading to discharge. He re-presented after 12 hours, and repeat ECG demonstrated evolution to a Wellen pattern. Coronary angiography showed a critical mid-LAD lesion with preserved distal flow and a near-occlusive mid–right coronary artery (RCA) lesion with collateral-dependent distal RCA filling from the contralateral side. PCI to the RCA resulted in abrupt LAD artery closure, with angiographic evidence of collateral flow reversal and distal LAD filling from the RCA.

**Discussion:**

This case illustrates dynamic ECG evolution in LAD ischemia and pressure-dependent bidirectional coronary collateral flow.

**Take-Home Messages:**

A de Winter ECG may evolve into Wellens pattern in critical LAD disease. Dynamic collaterals may influence PCI strategy in acute coronary syndrome.

## Visual Summary

**Figure:**
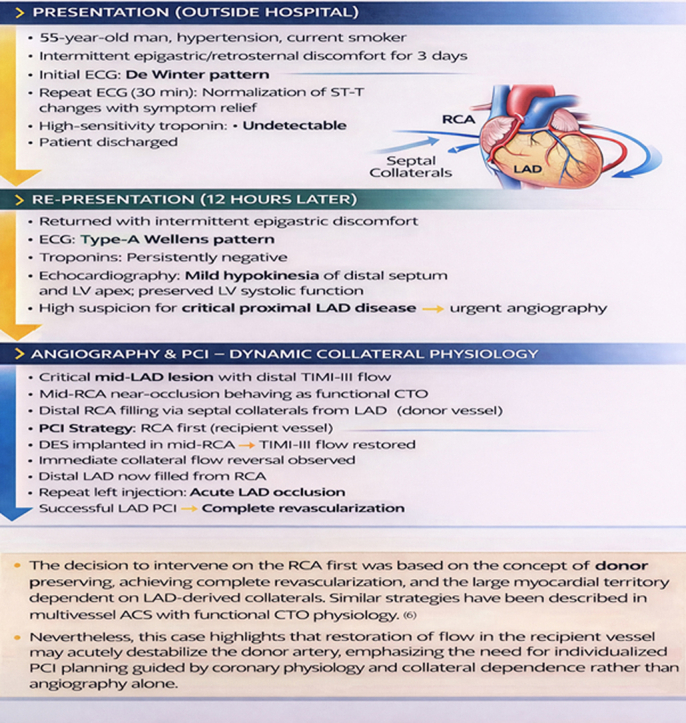

**Visual Summary:**
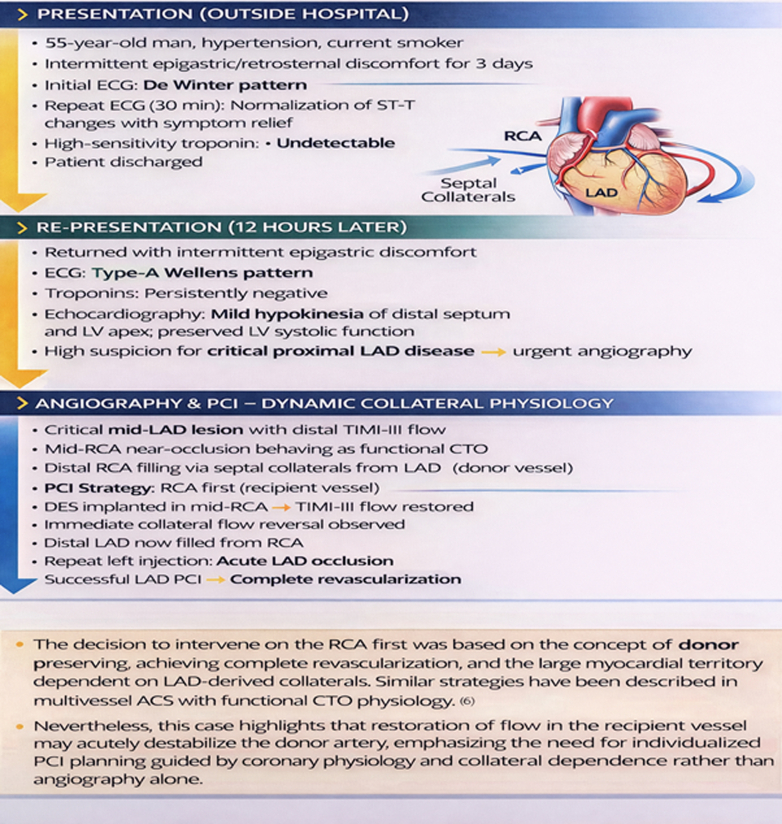
Timeline of Case Presentation ACS = acute coronary syndrome; CTO = chronic total occlusion; DES = drug-eluting stent; ECG = electrocardiogram; LAD = left anterior descending artery; LV = left ventricle; PCI = percutaneous coronary intervention; RCA = right coronary artery.

## History of Presentation

**Figure 1:**
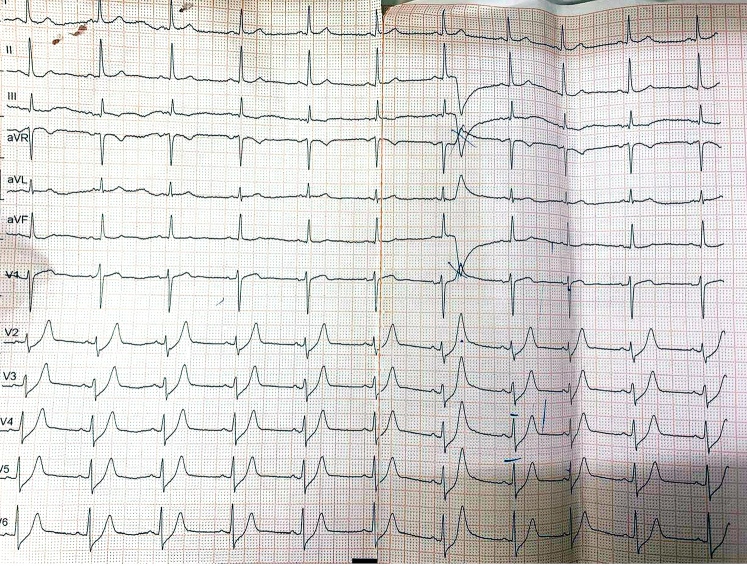
Electrocardiogram Obtained at Presentation Demonstrating Upsloping ST-Segment Depression With Tall, Symmetric T Waves in the Precordial Leads, Consistent With the de Winter Pattern The de Winter pattern is a STEMI equivalent indicative of acute proximal left anterior descending artery ischemia. STEMI = ST-segment elevation myocardial infarction.

**Figure 2:**
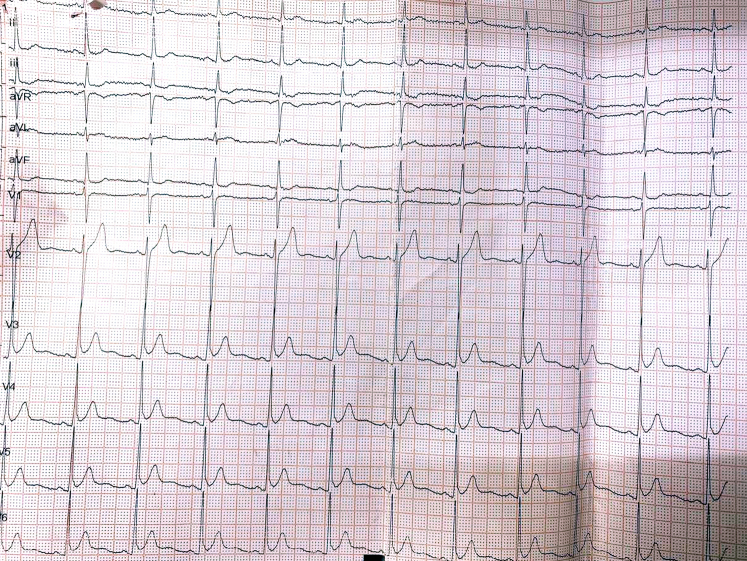
Repeat Electrocardiogram Obtained During Emergency Department Observation Demonstrating Near-Complete Normalization of ST Segments and T Waves

**Figure 3:**
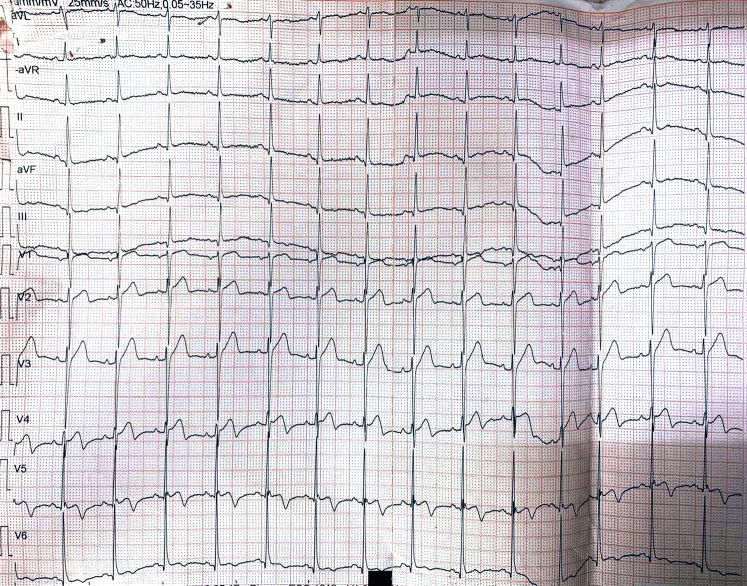
Electrocardiogram on Second Visit 12 Hours After First ECG Showing Biphasic T Waves in Leads V_2_ and V_3_, Consistent With Type A Wellens Syndrome Indicating a Critical but Transiently Patent LAD Lesion With High Risk of Imminent Occlusion ECG = electrocardiogram; LAD = left anterior descending artery.

A 55-year-old man and current smoker initially presented to an outside hospital with intermittent retrosternal discomfort of 2 days' duration. The initial electrocardiogram (ECG) demonstrated a de Winter pattern (Figure 1), characterized by upsloping ST-segment depression with tall, symmetrical T waves in the precordial leads. High-sensitivity cardiac troponin I levels were below the limit of detection. The patient was managed in the emergency department, and a repeat ECG (Figure 2) obtained approximately 30 minutes later showed near-complete resolution of the ST-T abnormalities, accompanied by symptomatic improvement. He was subsequently discharged. Twelve hours later, he presented to our hospital with mild epigastric discomfort. The ECG on arrival demonstrated a type A Wellens pattern (Figure 3), with biphasic T waves in the anterior precordial leads. Serial high-sensitivity troponin measurements remained below threshold levels for detection. Symptoms were intermittent, and the patient remained hemodynamically stable with normal vital signs. General physical and systemic examinations were unremarkable.

**Take-Home Messages**

• The de Winter and Wellens patterns represent a dynamic continuum of critical LAD ischemia and should be recognized as high-risk STEMI equivalents, even in the absence of biomarker elevation.
• Serial ECGs are essential, as transient normalization may falsely reassure and delay definitive management.
• Collateral circulation in multivessel ACS is dynamic and bidirectional: Changes in one vascular territory can abruptly alter flow in another.
• Revascularization of the collateral-dependent (“recipient”) vessel may destabilize the donor artery; thus, PCI strategy should be individualized, integrating coronary physiology, collateral dependence, and myocardial territory at risk rather than relying on angiography alone.

## Past Medical History

The patient had a history of hypertension, managed with telmisartan and amlodipine for the past 10 years. There was no prior history of diabetes mellitus or manifest coronary artery disease.

## Differential Diagnosis

Given the dynamic ECG changes and recurrent symptoms, the primary diagnosis was acute coronary syndrome (ACS) (unstable angina) with ECG evolution from de Winter pattern to Wellens syndrome, suggestive of a critically stenosed but transiently patent left anterior descending artery (LAD).

## Investigations

**Figure 4:**
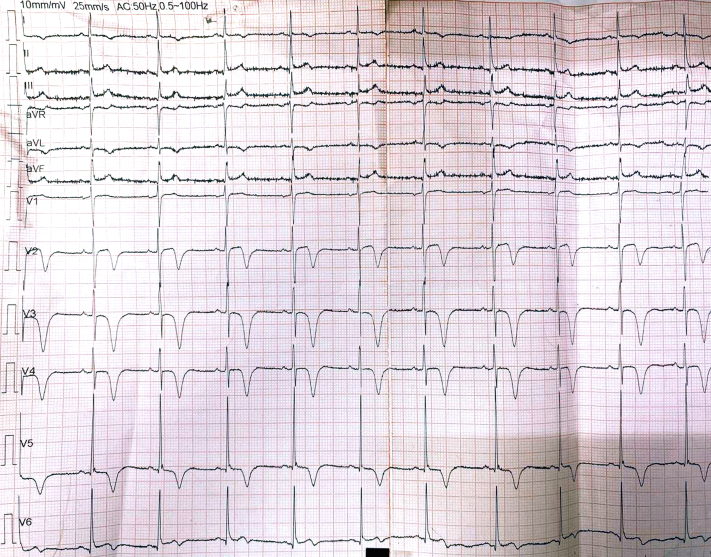
Electrocardiogram Immediately After Revascularization Showing Symmetrical Deep T-Wave Inversion in the Anterior Chest Leads, Indicative of Successful Reperfusion of LAD Territory LAD = left anterior descending artery.

Serial ECG findings were as follows:

• Initial ECG (Figure 1): de Winter pattern with upsloping ST-segment depression >1 mm at the J point in leads V_2_ to V_6_, tall symmetric T waves, and mild ST-segment elevation in lead aVR.
• Repeat ECG after 30 minutes (Figure 2): normalization of ST-T changes.
• ECG at second presentation (Figure 3): biphasic T waves in leads V_2_ to V_4_ consistent with type A Wellens syndrome.
• No significant ECG changes were observed during the procedure.
• Postrevascularization ECG (Figure 4): deep symmetrical T-wave inversion in the anterior leads, consistent with reperfusion.

High-sensitivity cardiac troponin I was measured serially and remained below threshold levels for detection.

Transthoracic echocardiography performed just before catheterization showed mild hypokinesia of the distal interventricular septum and left ventricular apex with preserved global systolic function. Left ventricular wall thickness and chamber sizes were normal. Mild concentric left ventricular hypertrophy with grade I diastolic dysfunction was noted.

## Management

The patient was treated as having ACS, admitted to the cardiac care unit, and received standard treatment in the form of dual antiplatelet therapy (aspirin 325 mg and ticagrelor 180 mg), high-intensity statin therapy (atorvastatin 80 mg), and anticoagulation (enoxaparin 60 mg). In view of dynamic ischemic ECG changes and high suspicion for critical LAD disease, he was shifted for urgent coronary intervention.

### Coronary angiography

**Figure 5:**
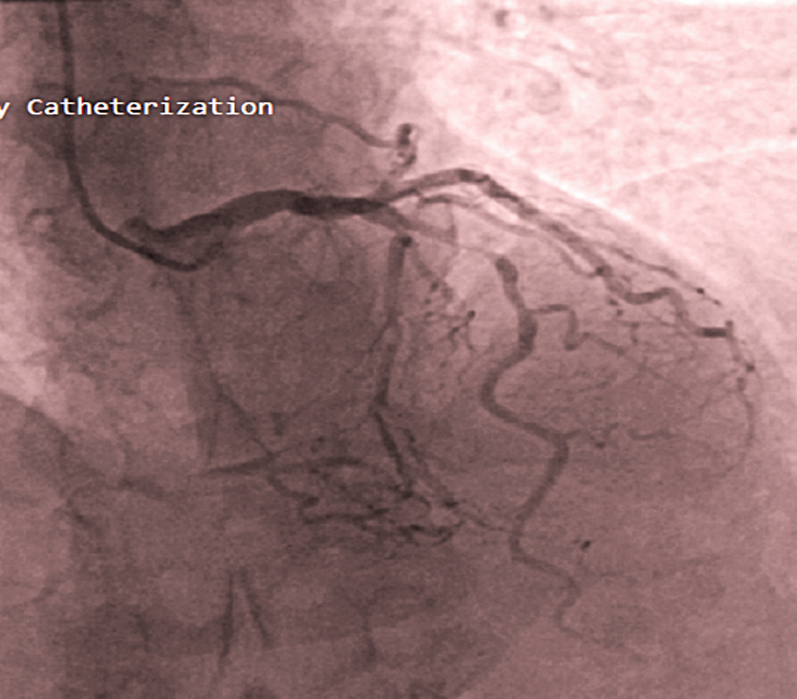
Coronary Angiography Showing Left Coronary System Critically Stenosed Mid-LAD Artery and Distal RCA Filling Through Septal Collaterals From the LAD LAD = left anterior descending artery; RCA = right coronary artery.
• Severe mid–right coronary artery (RCA) stenosis (subtotal occlusion) with distal RCA and posterior descending artery filling predominantly via septal collaterals from the LAD (Figure 6, Video 1b and Video 2a).

Angiography revealed the following:

• Severe (≈95%) stenosis in the mid-LAD with preserved antegrade distal flow (Figure 5, Video 1a).

**Figure 6:**
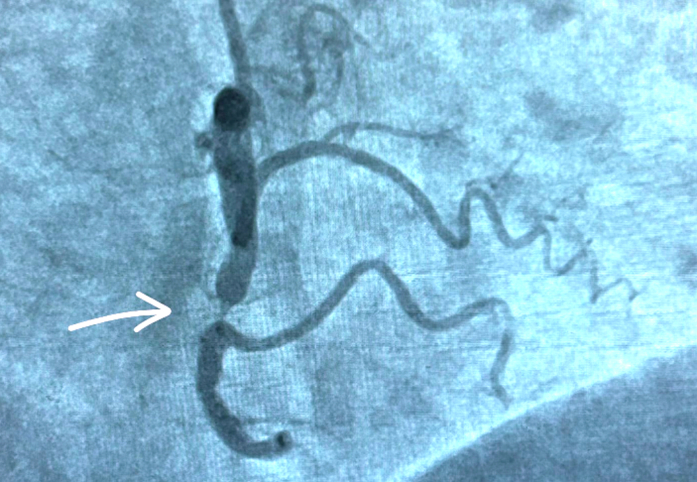
Coronary Angiography Showing Mid-RCA Tight Stenosis (Subtotal Occlusion; Arrow) With Slow Flow in the Distal RCA RCA = right coronary artery.
• Antegrade RCA injections showed poor distal opacification with TIMI flow grade II (Video 2b).

These findings suggested that the LAD was functioning as the dominant donor vessel, supplying a large myocardial territory including the distal RCA circulation. The mid RCA subtotal occlusion contributed to ongoing ischemic burden and was a significant challenge to the percutaneous coronary intervention (PCI) strategy.

### Percutaneous coronary intervention

**Figure 7:**
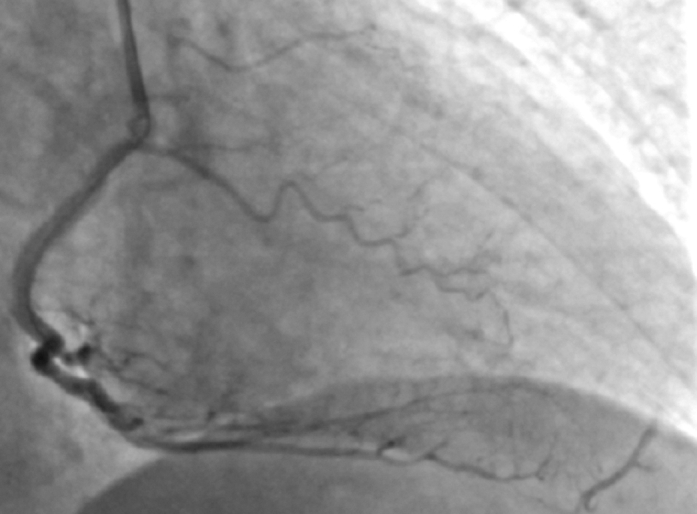
Coronary Angiography Showing Distal LAD Filling From the RCA and Flow Reversal in the Collaterals LAD = left anterior descending artery; RCA = right coronary artery.

**Figure 8:**
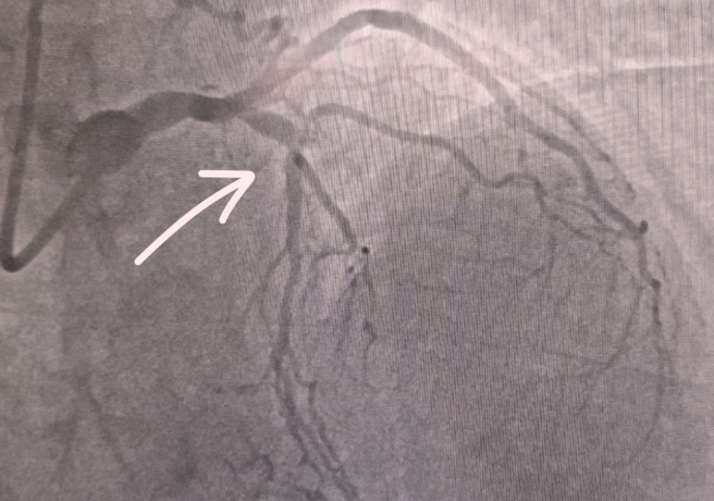
Coronary Angiography Showing Abrupt Closure of the LAD (Arrow) Due to Competitive Flow After PCI to the RCA LAD = left anterior descending artery; PCI = percutaneous coronary intervention; RCA = right coronary artery.

With the intention of complete revascularization and given the extensive collateral dependence of the dominant distal RCA territory, PCI to the RCA was performed first. After successful restoration of brisk antegrade RCA flow, repeat angiography demonstrated flow reversal in the collaterals (Figure 7, Video 3), with retrograde filling of the distal LAD from the RCA. Shortly thereafter, left coronary injection revealed acute occlusion of the previously patent critical mid-LAD lesion (Figure 8, Video 4). There were no significant hemodynamic or electrocardiographic changes observed during this critical juncture, as the patient was continuously monitored for any new symptom or event. Immediate PCI to the LAD was performed, restoring TIMI flow grade III with an excellent final angiographic result (Video 5). The patient remained hemodynamically stable throughout the procedure and was transferred to the cardiac care unit with stable vital parameters.

## Outcome and Follow-Up

The patient had an uneventful postprocedural course. Complete revascularization was achieved, with good angiographic and clinical outcomes. He was discharged on day 2 and remained asymptomatic on follow-up.

## Discussion

This case demonstrates the dynamic ECG and hemodynamic behavior of critical LAD disease in the setting of ACS. The evolution from a de Winter pattern to transient normalization and subsequently to Wellens syndrome reflects fluctuating coronary perfusion across a critically stenosed LAD segment, consistent with previously described preinfarction instability of the LAD artery. The de Winter and Wellens patterns are recognized as STEMI (ST-segment elevation myocardial infarction) equivalents and are associated with a high risk of imminent anterior myocardial infarction if not promptly revascularized.^1^, ^2^, ^3^

Coronary collateral circulation has traditionally been considered a static and unidirectional compensatory mechanism supplying chronically ischemic myocardium. However, both experimental and clinical studies have demonstrated that collateral flow is pressure dependent, recruitable, and capable of rapid reversal in response to changes in coronary hemodynamics.^4^^,^^5^ In the present case, the LAD functioned as the dominant donor vessel supplying the distal RCA territory through septal collaterals, thereby maintaining a favorable distal pressure gradient across a critically stenosed LAD lesion. Revascularization of the RCA resulted in abrupt restoration of distal RCA pressure, leading to loss of the LAD-to-RCA pressure gradient and sudden reversal of collateral flow. This hemodynamic shift caused a reduction in distal LAD run-off, precipitating acute occlusion of the previously patent critical LAD lesion. Such interdependence between coronary territories has been described in multivessel disease and functional chronic total occlusion physiology, where vessel patency may depend on balanced collateral-derived pressure support.^6^^,^^7^ This phenomenon, which we describe as “coronary collateral flow-reversal phenomenon,” differs from classic coronary steal. Instead, it represents a dynamic redistribution of coronary pressures resulting in destabilization of a critically stenosed donor vessel. Recognition of this mechanism is clinically important, as PCI sequencing in complex multivessel ACS can have unanticipated consequences when one vessel is heavily collateral dependent.

### Rationale for revascularization strategy

The decision to intervene on the RCA first was guided by the donor-vessel preservation concept, goals of achieving complete revascularization, and protecting the large myocardial territory supported by LAD-derived collaterals. Similar approaches have been described in multivessel ACS with functional chronic total occlusion physiology.^6^

This case highlights several important considerations:

• Restoration of antegrade flow in the recipient vessel can acutely destabilize the donor artery.
• Collateral flow reversal may precipitate sudden donor-vessel occlusion.
• PCI strategy in multivessel ACS should be individualized.
• Decision-making should be guided by coronary physiology and collateral dynamics rather than angiography alone.

## Conclusions

Coronary collateral circulation in ACS may be dynamic, bidirectional, and pressure dependent. Revascularization of a collateral-dependent vessel can abruptly alter coronary hemodynamics, resulting in acute occlusion of a critically stenosed donor artery.

## Funding Support and Author Disclosures

The authors have reported that they have no relationships relevant to the contents of this paper to disclose.

## Appendix

Video 1 Left coronary injection showing critical mid LAD lesion with preserved distal flow. (A) Left coronary system injection showing mid LAD artery critical stenosis, normal distal opacification. (B) Left coronary system injection showing distal RCA filling through septal collaterals from LAD artery.

Video 2 Left coronary injection showing collateral dependent distal RCA filling from LAD through septal collaterals. (A) Right coronary injection showing tight focal midsegment stenotic lesion. (B) Right coronary injection showing poor opacification of distal RCA.

Video 3 Right Coronary Injection Showing Flow Reversal in the Collaterals and LAD Filling From the RCA After PCI to the RCA

Video 4 Left Coronary System Injection After PCI to the RCA Showing Abrupt Closure of the LAD Artery

Video 5 Left Coronary Injection Showing the Final Result After PCI to the LAD

## References

[1] de Winter R.J., Verouden N.J.W., Wellens H.J.J., Wilde A.A.M., Interventional Cardiology Group of the Academic Medical Centre (2008). A new ECG sign of proximal LAD occlusion. N Engl J Med.

[2] Gorgels A.P.M., Engelen D.J.M., Wellens H.J.J. (2023). Pre-infarction electrocardiographic patterns of left anterior descending artery occlusion: contemporary recognition and management. JACC Case Rep.

[3] Meyers H.P., Bracey A., Lee D. (2023). STEMI equivalents: contemporary electrocardiographic recognition of acute coronary occlusion. J Am Coll Cardiol.

[4] Tajti P., Karmpaliotis D., Alaswad K. (2023). Contemporary outcomes of chronic total occlusion percutaneous coronary intervention: insights from a multicentre registry. JACC Cardiovasc Interv.

[5] Seiler C. (2003). The human coronary collateral circulation. Heart.

[6] Virani S.S., Newby L.K., Arnold S.V., Writing Committee Members (2023). 2023 AHA/ACC guideline for the management of chronic coronary disease. J Am Coll Cardiol.

[7] Brilakis E.S., Grantham J.A., Rinfret S. (2012). A percutaneous treatment algorithm for crossing coronary chronic total occlusions. JACC Cardiovasc Interv.

